# Predictors of Postpartum Depression in Korean Women: A National Cross-Sectional Study During the COVID-19 Pandemic

**DOI:** 10.3390/healthcare13101128

**Published:** 2025-05-12

**Authors:** Myongsun Cho, Meen Hye Lee

**Affiliations:** Department of Nursing, Gangneung-Wonju National University, Wonju-si 26403, Republic of Korea; mscho@gwnu.ac.kr

**Keywords:** COVID-19, postpartum depression, social isolation, risk factors, South Korea

## Abstract

Background/Objectives: Postpartum depression (PPD) affects maternal well-being and infant development, with the COVID-19 pandemic exacerbating mental health challenges for new mothers. This study examined the prevalence and predictors of PPD among Korean mothers in the early postpartum period. Methods: A nationwide cross-sectional study was conducted in South Korea from 10 September 2021. A two-stage stratified cluster sampling method recruited 3127 mothers who gave birth between January and December 2020. Data collection included the Edinburgh Postnatal Depression Scale (EPDS) and assessments of sociodemographic, pregnancy-related, infant health, and paternal involvement factors. Results: The prevalence of PPD (EPDS score ≥ 13) was 26.5%. Risk factors included a history of depression during pregnancy (OR = 8.65, *p* < 0.001), multiparity (OR = 1.03, *p* = 0.012), and frequent infant medical treatments (OR = 1.04, *p* < 0.001). Protective factors included better maternal health (OR = 0.36, *p* < 0.001), longer postpartum care (OR = 0.99, *p* < 0.001), enhanced postpartum education (OR = 0.97, *p* < 0.001), positive infant health perception (OR = 0.44, *p* < 0.001), and greater paternal involvement (OR = 0.97, *p* < 0.001). Conclusions: PPD is influenced by multiple factors, including maternal health, infant well-being, and paternal support. Routine screening and tailored interventions are essential to improve maternal mental health. Policies promoting holistic postpartum care and paternal involvement should be prioritized, especially during global crises like COVID-19.

## 1. Introduction

The World Health Organization (WHO) defines the postpartum period as the first six weeks (42 days) following childbirth, recognizing it as a critical time for mothers, newborns, parents, caregivers, and families. The WHO guidelines emphasize postpartum care services as an essential component of maternal and newborn health management, aiming to establish healthy practices, prevent complications, detect and manage illnesses, and ultimately enhance maternal and infant well-being [1].

Postpartum depression (PPD) is a major depressive disorder occurring after childbirth, with symptoms ranging from sadness and irritability to impaired maternal–infant bonding. The DSM-5 defines PPD as a major depressive episode with peripartum onset, typically within the first four weeks after delivery [2]. Globally, the prevalence of PPD is estimated at 17.7%, with higher rates in low- and middle-income countries [3,4]. In South Korea, a large-scale study involving 80,116 women who participated in the Seoul Healthy First Step Program (SHFSP) reported that 17.7% were at risk for peripartum depression, with 14.2% experiencing prepartum depression and 24.3% experiencing postpartum depression [5]. Studies have indicated that the prevalence of PPD increased during the COVID-19 pandemic. Guvenc et al. reported that the prevalence of PPD during the pandemic was as high as 34%, largely due to fear of infection and pandemic-related stressors [6].

Various factors contribute to the development of postpartum depression (PPD), and these determinants have become increasingly complex during the COVID-19 pandemic. Broadly, the risk factors for PPD can be categorized into sociodemographic characteristics, pregnancy- and childbirth-related conditions, and psychosocial domains. Sociodemographic characteristics such as marital status, socioeconomic status, and self-esteem have been found to be associated with PPD in several studies [7]. However, some evidence suggests that variables such as income level and educational attainment do not consistently predict the onset of PPD [8], highlighting the need for further investigation. In addition to sociodemographic influences, pregnancy- and childbirth-related conditions—such as unplanned pregnancies, cesarean deliveries, and obstetric complications—are linked to an increased risk of PPD, often due to heightened emotional distress and a reduced sense of control during labor [9]. The health condition of the infant, especially when medical complications or neonatal intensive care unit (NICU) admission is involved, is another important contributor to maternal psychological distress, including symptoms of anxiety and depression [10]. Moreover, paternal involvement in childcare has emerged as a key contextual factor. Supportive and active participation from fathers can help reduce maternal stress and serve as a protective factor against PPD, whereas limited engagement may exacerbate maternal feelings of isolation and increase the likelihood of depressive symptoms [11].

Postpartum depression (PPD) is a significant mental health concern that affects women after childbirth and has profound effects on their physical and psychological well-being. PPD is characterized by symptoms such as sadness, lethargy, hopelessness, insomnia, and difficulty concentrating and may be exacerbated by inadequate postpartum mental health management, leading to severe consequences [4,12]. During the postpartum period, a woman’s body undergoes critical physiological adjustments as it returns to its pre-pregnancy state, making this phase essential for maternal recovery. Beyond physical healing, the postpartum period is also pivotal for the formation and strengthening of maternal–newborn bonding, which plays a crucial role in the infant’s survival, growth, and development [13]. The establishment of a secure emotional attachment between mother and infant during this period significantly influences the newborn’s developmental outcomes, as well as the overall well-being of the family. Moreover, the continuity of affectionate maternal–infant bonding fosters essential cognitive and emotional development in the child, further highlighting the importance of adequate postpartum care and maternal mental health support [14]. Impairments in maternal responsiveness can hinder an infant’s emotional and social development, potentially resulting in emotional instability and developmental disorders in childhood [15]. In addition, PPD is associated with negative perceptions of parenting and low maternal self-esteem, which may have long-term effects on child health and development [16].

PPD has emerged as a critical social issue in South Korea. This development may be attributed to some unique sociocultural factors that affect postpartum women in South Korea. First, traditional gender roles and maternal ideologies in South Korea place significant expectations on women to fulfill the role of the perfect mother, often leading to increased stress and anxiety during the postpartum period. In addition, working mothers experience heightened psychological distress owing to conflicts between their careers and childcare roles, whereas non-working mothers frequently report feelings of guilt and anxiety related to intensive mothering expectations [17]. Considering the effects of these sociocultural factors, understanding the predictors of PPD within Korea’s unique postpartum care system is essential for developing effective postpartum mental health interventions tailored to postpartum women in the country [18].

Despite a growing body of literature examining individual risk factors for PPD, few studies have explored how these factors operate collectively within the unique context of the COVID-19 pandemic. In particular, the combined influence of sociodemographic characteristics, pregnancy, infant health-related conditions, and paternal involvement in childcare remains underexplored. Therefore, the aim of this study was to assess the prevalence of PPD among women in South Korea during the COVID-19 pandemic and to analyze the impact of maternal health and multidimensional factors on the development of PPD.

## 2. Materials and Methods

### 2.1. Study Design and Participants

**Figure 1:**
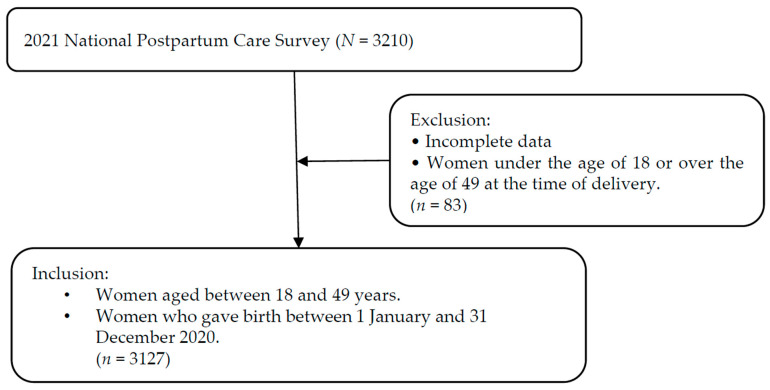
Flowchart of study participants.

This secondary analysis utilized data from the 2021 National Postpartum Care Survey, a nationally representative cross-sectional study conducted in South Korea under the Maternal and Child Health Act. The target population included women who gave birth between 1 January and 31 December 2020, and a total of 3127 participants were included in the final analysis after excluding those with missing data on the Edinburgh Postnatal Depression Scale (EPDS) (Figure 1).

According to national health guidelines, women of childbearing age are defined as those aged 18 to 49 years [19]. Therefore, the inclusion criteria were: (1) women aged 18–49 years at the time of childbirth, and (2) those who delivered during the year 2020. Participants were excluded if they fell outside this age range, did not give birth in 2020, had incomplete or implausible data, lived in hard-to-access areas, or did not consent to participate.

Data were collected between 1 and 10 September 2021, through both face-to-face and online methods using the same structured questionnaire, with the survey mode determined by participants’ preference.

The study was conducted in accordance with the guidelines of the Declaration of Helsinki and approved by the Institutional Review Board of G University (GWNUIRB-R2023-63; 21 September 2023).

### 2.2. Measurements

#### 2.2.1. Dependent Variable

PPD was assessed using the Edinburgh Postnatal Depression Scale (EPDS), a widely used self-report questionnaire that consists of 10 items that measure general depressive symptoms. Each item is scored on a scale of 0–3, with the total score ranging from 0 to 30. The Korean version of the EPDS (K-EPDS) was used in this study, with a conservative cutoff score of ≥13 to indicate PPD [20]. Results of studies on the validation of the K-EPDS suggest an optimal cutoff score of 10, with a sensitivity of 76.7% and specificity of 87.1% for detecting PPD. The reliability of the K-EPDS was demonstrated with a Cronbach’s α of 0.789 (0.765 in the present study).

Based on the information in the existing literature, the independent variables analyzed in this study included sociodemographic factors, pregnancy and childbirth-related factors, infant health status, and paternal involvement in childcare.

(1) Sociodemographic factors

The sociodemographic characteristics analyzed included region, maternal age, marital status, educational level, and household income. Maternal age was categorized into five groups: ≤24 years, 25–29 years, 30–34 years, 35–39 years, and ≥40 years. Educational level was classified as high school, college/university, and graduate school or higher. Household income was measured based on the average monthly income in million Korean won (KRW).

(2) Pregnancy and childbirth-related factors

The pregnancy and childbirth-related factors assessed in this study included pregnancy intention (planned/unplanned), parity (primipara/multipara), and duration of postpartum care (days). Additionally, maternal health management and infant care education were measured by evaluating whether the participants had received education on maternal health management, contraception, PPD management, infant care (including diapering, bathing, and umbilical cord care), infant safety, maternal safety, and breastfeeding. The total number of educational sessions attended was summed, with higher scores indicating greater exposure to educational support.

Maternal health status was assessed using a self-rated health measure in which participants were asked, “How would you rate your health in general?”. Responses were recorded on a 5-point Likert scale ranging from 1 (very poor) to 5 (very good) and were categorized into three groups: poor (“bad” or “very bad”), moderate (“fair”), and good (“very good” or “good”).

The perceived reasons for PPD were assessed using a 7-item scale, including factors such as the burden of childcare. Each item was rated on a 4-point Likert scale, ranging from 1 (“hardly had an impact”) to 4 (“had a very significant impact”), with higher scores indicating a greater influence on postpartum depressive symptoms.

#### 2.2.2. Independent Variables

(3) Infant health status

Infant health-related factors included maternal perceptions of infant health and the number of medical treatments received. Infant health perception was measured by asking the question, “How would you rate your baby’s health in general?”. Responses were recorded on a 5-point Likert scale with points ranging from 1 (very poor) to 5 (very good). The responses were classified as poor (“bad” or “very bad”), moderate (“fair”), and good (“very good” or “good”).

The number of infant medical treatments was assessed by recording the total number of times the infant received medical care for major health conditions, including respiratory infections, gastrointestinal issues, fever, umbilical complications, jaundice, tongue-tie surgery, accidental injuries, conjunctivitis, and other illnesses, within the first 12 months postpartum.

(4) Paternal involvement in childcare

Paternal involvement in childcare was assessed using an 8-item scale that measured the frequency of paternal participation in various childcare activities during the postpartum period. These activities included changing diapers, assisting with feeding, soothing the baby when crying, bathing the baby, playing with the baby, putting the baby to sleep, performing household chores, and caring for the other children. Each item was rated on a 5-point Likert scale ranging from 1 (very passive) to 5 (very active), with higher scores indicating greater paternal involvement in childcare. The internal consistency of this scale was validated with Cronbach’s α = 0.755.

### 2.3. Statistical Analysis

The objectives of this study were threefold: (1) to determine the prevalence of postpartum depression (PPD) among postpartum women in South Korea; (2) to compare sociodemographic characteristics, pregnancy and childbirth-related factors, infant health status, and spousal involvement in childcare between women with and without PPD; and (3) to identify the key factors associated with the development of PPD.

Descriptive statistics were used to summarize the characteristics of the study population. Chi-square tests were performed to assess group differences in categorical variables between women with and without PPD. To identify factors independently associated with PPD experiences within the past 12 months, hierarchical logistic regression modeling was conducted. Adjusted odds ratios (ORs) with 95% confidence intervals (CIs) were calculated. Statistical significance was set at *p* < 0.05. All analyses were performed using SPSS Statistics version 27.0 (IBM Corp., Armonk, NY, USA).

## 3. Results

### 3.1. Sociodemographic Factors

Of the 3127 postpartum women included in this study, 830 (26.5%) had PPD, whereas 2297 (73.5%) did not. Results of the comparative analysis of various factors associated with PPD in the study cohort, including sociodemographic factors, pregnancy-related variables, infant health factors, and paternal involvement in childcare, are shown in Table 1.

Women with PPD exhibited distinct sociodemographic and pregnancy-related characteristics compared to those without PPD. Specifically, women in the PPD group were significantly more likely to reside in urban areas (74.3% vs. 68.7%, *p* = 0.002), be single or unmarried (1.7% vs. 0.6%, *p* = 0.003), and have a lower level of education (high school vs. college/graduate, *p* = 0.040) than those in the non-PPD group (Table 1).

### 3.2. Self-Reported Contributing Factors of Postpartum Depression and Paternal Involvement in Childcare

The most frequently reported reason for experiencing PPD was the burden of childcare (1.76 ± 1.77), followed by stress due to environmental changes (1.70 ± 1.75) and maternal physical health after childbirth (1.67 ± 1.73). Changes in body weight and physical appearance (1.57 ± 1.67) and lack of support from the spouse and family (1.34 ± 1.49) were also identified as contributing factors. Interestingly, some women reported experiencing PPD without a specific reason (1.29 ± 1.48). Concerns regarding infant physical health (1.08 ± 1.27) were the least frequently reported cause of PPD.

Playing with the baby (3.80 ± 1.16) was the most commonly reported paternal childcare activity, followed by changing diapers (3.77 ± 1.22) and soothing the baby when crying (3.71 ± 1.19). Fathers participated in household chores (3.70 ± 1.25) and in bathing the baby (3.69 ± 1.38) at similar levels. Notably, assisting with feeding (3.50 ± 1.28) and putting the baby to sleep (3.45 ± 1.32) were reported less frequently than the abovementioned activities. Caring for the other children (1.88 ± 2.15) was reported least frequently, suggesting that fathers may be less engaged in supporting mothers with additional childcare responsibilities beyond caring for the newborn (Table 2).

### 3.3. Factors Associated with Postpartum Depression

Results of the logistic regression analysis of the factors associated with PPD are presented in Table 3. Model 1 included sociodemographic factors, which were controlled for in Model 2. Model 2 also included pregnancy- and childbirth-related factors, infant health status, and paternal involvement in childcare.

In Model 1, women residing in urban areas had a significantly higher risk of developing PPD than those living in rural areas (OR = 1.43, 95% CI: 1.41–1.47, *p* < 0.001). However, older maternal age, higher education level, and being married were protective factors against PPD (*p* < 0.001). Women with a monthly income of 3–4 million KRW had an increased risk of PPD (OR = 1.19, *p* < 0.001), whereas those with higher incomes (≥7 million KRW) showed a reduced risk (*p* < 0.001).

After controlling for sociodemographic factors in Model 2, additional pregnancy- and childbirth-related variables were significantly associated with the risk of PPD. Unplanned pregnancy was associated with an elevated likelihood of developing PPD (OR = 0.84, *p* < 0.001). A history of depression during pregnancy was the strongest predictor of PPD, increasing the risk of PPD 8.65-fold (*p* < 0.001). Women with better maternal health had significantly lower odds of developing PPD than those with worse maternal health, with moderate (OR = 0.60, *p* < 0.001) and good (OR = 0.36, *p* < 0.001) health ratings serving as protective factors against PPD. Longer duration of postpartum care was associated with a reduced risk of PPD (OR = 0.99, *p* < 0.001), whereas more frequent infant medical treatments increased the risk of PPD (OR = 1.04, *p* < 0.001). Additionally, better perceived infant health and greater paternal involvement in childcare were significantly associated with a reduced likelihood of developing PPD (OR = 0.97, *p* < 0.001) (Table 3).

## 4. Discussion

In this study, we used data from a national cohort of postpartum mothers in South Korea to analyze the prevalence of PPD and evaluate the impact of pregnancy-, childbirth-, and infant-related factors on PPD during the COVID-19 pandemic. The results showed that the prevalence of PPD during the COVID-19 pandemic was 26.5%, which is lower than the 34% reported in developed countries [21]. Notably, a systematic review indicated that the prevalence of PPD during the pandemic varied significantly, ranging from 6.4% to 56.9%, likely attributable to differences across populations and research methodologies [22]. These results consistently indicate that the post-pandemic prevalence of PPD is significantly higher than the pre-pandemic rates. This increased prevalence of PPD indicates that societal disruptions caused by global crises, such as the COVID-19 pandemic, have a profound impact on maternal mental health, particularly during the postpartum period.

The results of the present study indicate that social isolation is a significant risk factor for PPD. Restrictions on movement and reduced access to familial and social support networks during the pandemic exacerbated maternal stress and feelings of loneliness. These findings support those from previous studies that identified social isolation as a key determinant of PPD [9,23]. These effects of the limited availability of emotional and physical support during the pandemic highlight the urgent need for robust social support systems that can operate effectively to prevent PPD during crises. Virtual community-building programs and telehealth counseling services could help alleviate the impact of social isolation in comparable future pandemics [24].

In this study, we examined the multifaceted factors associated with PPD, including pregnancy-, childbirth-, and infant-related factors. Key demographic factors such as age, marital status, education level, income, and urban residency had a significant impact on the prevalence of PPD. Older mothers, those in stable marital relationships, and those with higher educational levels showed a lower prevalence of PPD than younger mothers, those who were unmarried, and those with lower educational levels. These findings suggest that emotional maturity, secure family dynamics, and improved access to information enhance coping abilities during the postpartum period. Additionally, higher education may support informed decision-making and effective problem-solving related to maternal and infant health [25,26].

Unexpectedly, urban residency and higher household income were associated with a higher prevalence of PPD. This indicates that urban residency may be associated with an increased risk of PPD. A previous study also showed that women living in urban areas with populations exceeding 500,000 exhibit a higher prevalence of PPD than those living in rural, semi-rural, or semi-urban areas [27]. Regarding income and PPD, findings on the association between income and PPD are inconsistent across studies. Although numerous previous studies have identified low income as a significant risk factor for PPD [4,28], the results of the present study suggest that financial stability alone may not be a direct predictor of PPD. Other stressors related to high-income households might play a role in the correlation between high income and the development of PPD. Similarly, a previous study conducted in Japan revealed no significant correlation between PPD and household income or maternal and paternal educational levels [29]. The results of the present study suggest that increased stress from urban living conditions and heightened childcare expectations in higher-income families contribute to the risk of PPD. Therefore, further studies are needed to clarify the complex dynamics of the relationship between urban stressors, financial pressure, and their impact on PPD.

Pregnancy- and childbirth-related factors have a substantial impact on PPD. The results of this study indicated that planned pregnancy and good maternal health significantly reduced the risk of PPD. These factors likely reflect better preparedness and resilience in managing postpartum challenges. Unintended pregnancies are associated with several adverse maternal health outcomes, including delayed prenatal care, reduced breastfeeding rates, increased maternal depression, and higher frequency of domestic abuse. A systematic review and meta-analysis revealed a significant association between unintended pregnancies and elevated rates of maternal depression during and after pregnancy, as well as a higher incidence of interpersonal violence [30]. Furthermore, a recent study emphasized that untreated PPD may result in long-term psychological effects, including anxiety disorders and persistent depressive symptoms, thereby affecting maternal health and family dynamics [31]. These studies underscore the critical need for early detection and intervention to mitigate the adverse effects of PPD on maternal health.

In this study, mothers with multiple births and those with a history of PPD exhibited higher rates of PPD. Findings from previous studies indicate that mothers who had multiple births have a higher risk of developing PPD than those who had singleton births. For example, mothers of multiples have 43% greater odds of experiencing moderate to severe depressive symptoms nine months postpartum than mothers of singletons [32]. Similarly, another study indicated that 69% of mothers of multiples experience stress, anxiety, and/or depression in the postnatal period, with over three-quarters feeling isolated [33]. These findings suggest that women who have given birth to multiple children may have an elevated risk of developing pregnancy anxiety and PPD, highlighting the need for targeted mental health interventions for multiparous mothers and those with known risk factors.

Infant health significantly influences maternal mental well-being. The results of this study indicated that mothers of healthier infants exhibited lower rates of PPD, whereas frequent hospital visits for infants were linked to an increased risk of PPD. This aligns with the existing evidence supporting an association between maternal mental health and infant health concerns. Findings from previous studies indicate that mothers of infants with health complications are at a higher risk of experiencing mental health issues, including PPD and anxiety, than those with healthier children. For example, mothers of infants admitted to neonatal intensive care units exhibit higher levels of depressive symptoms than mothers of healthy infants [34]. The impact of infant health issues on maternal mental well-being highlights the importance of establishing comprehensive support systems for affected mothers. In addition, integrated maternal–infant healthcare services that address both physical and psychological needs are necessary for tackling these challenges [35].

The results of the present study indicate that spousal involvement in childcare is a significant protective factor against PPD, which supports the evidence from previous studies. Research indicates that a partner’s active participation in caregiving activities enhances maternal mental health. Specifically, increased paternal involvement in infant care is associated with reduced maternal PPD symptoms [36]. In addition, supportive spousal relationships and active spousal involvement during the postpartum period are associated with decreased maternal depressive symptoms [37]. Childcare activities that the father could participate in include playing with the child, changing diapers, and comforting the baby, all of which are expected to help reduce maternal stress. The findings of the present study highlight the importance of promoting paternal participation in childcare through policies such as extended paternity leave and parenting education programs. Such initiatives could alleviate maternal burden and foster a more balanced distribution of childcare responsibilities. Taken together, the findings of this study are consistent with previous research on risk factors for postpartum depression (PPD), reinforcing the multifaceted nature of maternal mental health challenges [38].

The findings of this study have important implications for maternal mental health policies and practices. First, strengthening social support systems is crucial for reducing the prevalence of PPD, particularly during crises. Policymakers should invest in virtual community-building programs and telehealth counseling services to provide continuous support to postpartum women. In addition, healthcare providers should implement comprehensive postpartum support systems that address the childcare burden, environmental stressors, and physical recovery of new mothers. Targeted interventions, including educational programs and psychological support services, could help mitigate these risk factors [39]. Second, urban-specific stressors should be addressed through the development of tailored mental health programs. Local governments and healthcare providers should implement community-based interventions, such as parenting support groups and childcare assistance programs, to help reduce the psychological burden on mothers residing in urban areas. Additionally, policies and programs should encourage active paternal involvement in various childcare tasks to reduce maternal stress and lower the risk of PPD. Fathers’ engagement in newborn care and household responsibilities can play a crucial role in supporting maternal well-being during the postpartum period [39]. Finally, promoting spousal involvement through the implementation of workplace policies, such as extended paternity leave and parenting education initiatives, can enhance family dynamics and alleviate maternal stress. Encouraging equitable distribution of childcare responsibilities can play a significant role in reducing the burden of PPD and improving maternal well-being.

This study has some limitations. First, as this was a cross-sectional study, causality could not be determined. Future longitudinal research is required to explore the temporal progression of PPD and assess the long-term efficacy of targeted interventions. Second, the use of self-reported data may have introduced some potential bias into the findings. In particular, future research should aim to incorporate objective measures, including clinical data, to enhance the accuracy of findings. Third, the study was conducted during the COVID-19 pandemic—a period that may have intensified maternal health challenges due to elevated stress levels and limited access to healthcare services. Therefore, the findings should be interpreted with caution when extrapolated to non-pandemic contexts or other populations.

Despite these limitations, the study builds on the existing research by illustrating the compounded effects of pandemic-induced stressors on maternal mental health. Although previous studies have identified demographic and health-related factors as determinants of PPD, the findings of the present study uniquely highlight the role of societal disruptions, such as social isolation during the COVID-19 pandemic, in exacerbating these risks. This contextual difference emphasizes the importance of establishing adaptive healthcare systems capable of responding to extraordinary circumstances.

## 5. Conclusions

This study identified significant predictors of postpartum depression (PPD) among Korean mothers during the COVID-19 pandemic, revealing that PPD is influenced by a combination of sociodemographic, pregnancy-related conditions, infant health-related conditions, and paternal involvement in childcare. A history of depression during pregnancy, poor maternal health, unplanned pregnancy, frequent infant medical treatments, and low paternal involvement were found to be key risk factors. In contrast, higher maternal health ratings, longer durations of postpartum care, more infant care education sessions, perceived infant health, and greater paternal involvement were protective against PPD.

These findings highlight the necessity of implementing multidimensional interventions that address both physical recovery and emotional well-being during the postpartum period. Furthermore, the findings underscore the amplified impact of pandemic-related stressors—such as social isolation and limited access to healthcare—on maternal mental health. The elevated prevalence of PPD observed during the pandemic emphasizes the urgency of strengthening maternal health systems that can adapt to extraordinary public health emergencies. Policies promoting paternal engagement, virtual support platforms, and tailored postpartum education are essential to improving maternal outcomes. Future longitudinal studies are recommended to explore causal relationships and assess the long-term effectiveness of targeted interventions across diverse postpartum populations.

## Figures and Tables

**Table 1:** Comparison of sociodemographic factors between women with and without postpartum depression (*N* = 3127).

Factor		*N* (%)			*χ*^2^ or *t*	*p*-Value
		Total	No PPD (*N* = 2297)	PPD (*N* = 830)		
Region	Rural	933 (29.8%)	720 (31.3%)	213 (25.7%)	9.41	0.002
	Urban	2194 (70.2%)	1577 (68.7%)	617 (74.3%)		
Age	≤24 years	68 (2.2%)	44 (1.9%)	24 (2.9%)	5.8	0.215
	25–29 years	422 (13.5%)	301 (13.1%)	121 (14.6%)		
	30–34 years	1248 (39.9%)	938 (40.8%)	310 (37.3%)		
	35–39 years	1072 (34.3%)	780 (34.0%)	292 (35.2%)		
	40–49 years	317 (10.1%)	234 (10.2%)	83 (10.0%)		
Marital status	Single/unmarried	27 (0.9%)	13 (0.6%)	14 (1.7%)	8.95	0.003
	Married	3100 (99.1%)	2284 (99.4%)	816 (98.3%)		
Level of education	High school	502 (16.1%)	346 (15.1%)	156 (18.8%)	6.42	0.040
	College/University	2327 (74.4%)	1732 (75.4%)	595 (71.7%)		
	Graduate school and above	298 (9.5%)	219 (9.5%)	79 (9.5%)		
Average monthly income (million KRW)	1–2	155 (5%)	111 (4.8%)	44 (5.3%)	6.58	0.160
	3–4	1551 (49.6%)	1112 (48.4%)	439 (52.9%)		
	5–6	921 (29.5%)	696 (30.3%)	225 (27.1%)		
	7–8	351 (11.2%)	262 (11.4%)	89 (10.7%)		
	Greater than 8	149 (4.8%)	116 (5.1%)	33 (4.0%)		
Pregnancy plan	Unplanned	1125 (36.0%)	790 (34.4%)	335 (40.4%)	9.43	0.002
	Planned	2002 (64.0%)	1507 (65.6%)	495 (59.6%)		
Parity	Primipara	1715 (54.8%)	1245 (54.2%)	470 (56.6%)	1.45	0.229
	Multipara	1412 (45.2%)	1052 (45.8%)	360 (43.4%)		
Depression in pregnancy	No depression	1497 (47.9%)	1390 (60.5%)	107 (12.9%)	554.08	<0.001
	Depression	1630 (52.1%)	907 (39.5%)	723 (87.1%)		
Maternal health status		3.14 ± 0.98	3.31 ± 0.93	2.66 ± 0.97	17.26	<0.001
Duration of postpartum care		30.15 ± 21.46	31.47 ± 21.23	26.5 ± 21.66	5.75	<0.001
Number of infant care education sessions		3.03 ± 2.38	3.12 ± 2.42	2.79 ± 2.25	3.58	<0.001
Number of infant medical treatments		1.03 ± 1.02	4.32 ± 0.78	4.09 ± 0.9	6.55	<0.001
Perceived health of the infant		4.26 ± 0.82	0.97 ± 0.98	1.2 ± 1.1	−5.42	<0.001
Paternal involvement in childcare		27.52 ± 7.76	28.29 ± 7.27	25.36 ± 8.62	8.75	<0.001

Note. PPD, postpartum depression.

**Table 2:** Self-reported contributing factors of postpartum depression and paternal involvement in childcare.

Self-Reported Contributing Factors of PPD	M ± SD
Burden of childcare	1.76 ± 1.77
Stress due to environmental changes	1.70 ± 1.75
Maternal physical health after childbirth	1.67 ± 1.73
Changes in body weight and physical appearance	1.57 ± 1.67
Lack of support from the spouse and family	1.34 ± 1.49
No specific reason	1.29 ± 1.48
Infant’s physical health condition	1.08 ± 1.27
Paternal involvement in childcare	M ± SD
Playing with the baby	3.80 ± 1.16
Changing diapers	3.77 ± 1.22
Soothing the baby when crying	3.71 ± 1.19
Performing household chores	3.70 ± 1.25
Bathing the baby	3.69 ± 1.38
Assisting with feeding	3.50 ± 1.28
Putting the baby to sleep	3.45 ± 1.32
Caring for the other children	1.88 ± 2.15

M = mean, SD = standard deviation.

**Table 3:** Logistic regression analysis of factors associated with postpartum depression.

Factor		Model 1	*p*-Value	Model 2	*p*-Value
		95% CI		95% CI	
Region	Urban (ref = rural)	1.43 (1.4–1.47)	<0.001	1.29 (1.25–1.33)	<0.001
Age (ref = ≤24 years)	≤24 years	0.67 (0.63–0.71)	<0.001	0.70 (0.66–0.75)	<0.001
	30–34 years	0.64 (0.61–0.68)	<0.001	0.63 (0.59–0.67)	<0.001
	35–39 years	0.68 (0.64–0.71)	<0.001	0.66 (0.62–0.71)	<0.001
	40–49 years	0.69 (0.65–0.73)	<0.001	0.62 (0.58–0.66)	<0.001
Marital status (ref = unmarried)	Married	0.30 (0.28–0.33)	<0.001	0.65 (0.58–0.72)	<0.001
Level of education (ref = high school graduate)	College/University	0.81 (0.79–0.83)	<0.001	0.69 (0.67–0.71)	<0.001
	Graduate school and above	0.76 (0.73–0.79)	<0.001	0.60 (0.58–0.63)	<0.001
Average monthly income (ref = 1–2 million KRW)	3–4	1.19 (1.14–1.25)	<0.001	1.45 (1.38–1.53)	<0.001
	5–6	1.02 (0.98–1.07)	0.329	1.39 (1.32–1.47)	<0.001
	7–8	0.87 (0.82–0.91)	<0.001	1.20 (1.14–1.28)	<0.001
	Greater than 8	1.02 (0.97–1.08)	0.453	1.48 (1.38–1.58)	<0.001
Planned pregnancy (ref = unplanned)				0.84 (0.83–0.86)	<0.001
Multipara (ref = primipara)				1.03 (1.01–1.05)	0.012
Depression in pregnancy (ref = no depression)				8.65 (8.44–8.87)	<0.001
Maternal health status (ref = Poor)	Moderate			0.60 (0.59–0.62)	<0.001
	Good			0.36 (0.35–0.37)	<0.001
Duration of postpartum care				0.99 (0.99–0.99)	<0.001
Number of infant care education sessions				0.97 (0.97–0.98)	<0.001
Number of infant medical treatments				1.04 (1.03–1.05)	<0.001
Perceived health of the infant (ref = Poor)	Moderate			0.80 (0.76–0.85)	<0.001
	Good			0.44 (0.42–0.46)	<0.001
Paternal involvement in childcare				0.97 (0.97–0.98)	<0.001

CI = confidence interval.

## Data Availability

The original data presented in the study are openly available in MicroData Integrated Service at https://mdis.kostat.go.kr/dwnlSvc/ofrSurvSearch.do?curMenuNo=UI_POR_P9240 on 3 June 2023.

## References

[1] WHO Recommendations on Maternal and Newborn Care for a Positive Postnatal Experience. https://www.who.int/publications/i/item/9789240045989.

[2] American Psychiatric Association (2013). Diagnostic and Statistical Manual of Mental Disorders.

[3] Hahn-Holbrook J., Cornwell-Hinrichs T., Anaya I. (2017). Economic and Health Predictors of National Postpartum Depression Prevalence: A Systematic Review, Meta-Analysis, and Meta-Regression of 291 Studies From 56 Countries. Front. Psychiatry.

[4] Mitchell A.R., Gordon H., Lindquist A., Walker S.P., Homer C.S.E., Middleton A., Cluver C.A., Tong S., Hastie R. (2023). Prevalence of Perinatal Depression in Low- and Middle-Income Countries: A Systematic Review and Meta-Analysis. JAMA Psychiatry.

[5] Kang S.Y., Khang Y.-H., June K.J., Cho S.-H., Lee J.Y., Kim Y.-M., Cho H.-J. (2022). Prevalence and Risk Factors of Maternal Depression among Women Who Participated in a Home Visitation Program in South Korea. Soc. Psychiatry Psychiatr. Epidemiol..

[6] Guvenc G., Yesilcinar İ., Ozkececi F., Öksüz E., Ozkececi C.F., Konukbay D., Kok G., Karasahin K.E. (2021). Anxiety, Depression, and Knowledge Level in Postpartum Women During the COVID-19 Pandemic. Perspect. Psychiatr. Care.

[7] Kovacheva K., Rodríguez-Muñoz M.F., Gómez-Baya D., Domínguez-Salas S., Motrico E. (2023). The Socio-Demographic Profile Associated With Perinatal Depression During the COVID-19 Era. BMC Public Health.

[8] Szurek-Cabanas R., Navarro-Carrillo G., Martínez-Sánchez C.A., Oyanedel J.C., Villalobos D. (2024). Socioeconomic Status and Maternal Postpartum Depression: A PRISMA-Compliant Systematic Review. Curr. Psychol..

[9] Sahebi A., Kheiry M., Abdi K., Qomi M., Golitaleb M. (2024). Postpartum Depression During the COVID-19 Pandemic: An Umbrella Review and Meta-Analyses. Front. Psychiatry.

[10] Beck D.C., Tabb K.M., Tilea A., Hall S.V., Vance A., Patrick S.W., Schroeder A., Zivin K. (2022). The Association Between NICU Admission and Mental Health Diagnoses Among Commercially Insured Postpartum Women in the US, 2010–2018. Children.

[11] Wang D., Li Y.-L., Qiu D., Xiao S.-Y. (2021). Factors Influencing Paternal Postpartum Depression: A Systematic Review and Meta-Analysis. J. Affect. Disord..

[12] Bennett H.A., Einarson A., Taddio A., Koren G., Einarson T.R. (2004). Prevalence of Depression During Pregnancy: Systematic Review. Obstet. Gynecol..

[13] Bystrova K., Ivanova V., Edhborg M., Matthiesen A.-S., Ransjö-Arvidson A.-B., Mukhamedrakhimov R., Uvnäs-Moberg K., Widström A.-M. (2009). Early Contact Versus Separation: Effects on Mother-Infant Interaction One Year Later. Birth.

[14] Murray L., Stanley C., Hooper R., King F., Fiori-Cowley A. (1996). The Role of Infant Factors in Postnatal Depression and Mother-Infant Interactions. Dev. Med. Child Neurol.

[15] Field T. (2010). Postpartum Depression Effects on Early Interactions, Parenting, and Safety Practices: A Review. Infant Behav. Dev..

[16] Beck C.T. (1995). The Effects of Postpartum Depression on Maternal-Infant Interaction: A Meta-Analysis. Nurs. Res..

[17] Kim S., Yoo J., Seol K.O. (2023). The Mediating Effects of Role Orientation and the Meaning of Life on the Relationship between Mother’s Intensive Motherhood Ideology and Life Satisfaction: Role Orientation and Meaning of Life as Mediators, and Multi-group Analysis Based on Employment Status. J. Soc. Sci..

[18] Kim M.N., Choi S.Y. (2013). A Comparative Study of Postpartum Stress, Postpartum Depression, Postpartum Discomfort and Postpartum Activity, Between Women Who Used and Those Women Did Not Used Sanhujori Facilities. J. Korean Soc. Matern Child Health.

[19] Liu X., Wang S., Wang G. (2021). Prevalence and Risk Factors of Postpartum Depression in Women: A Systematic Review and Meta-analysis. J. Clin. Nurs..

[20] Ahn C.S., Kang M.S., Park S.Y., Choi Y.R. (2015). Usefulness of Edinburgh Postnatal Depression Scale for Postpartum Depression. Korean J. Perinatol..

[21] Chen Q., Li W., Xiong J., Zheng X. (2022). Prevalence and Risk Factors Associated With Postpartum Depression During the COVID-19 Pandemic: A Literature Review and Meta-Analysis. Int. J. Environ. Res. Public Health.

[22] Behera D., Bohora S., Tripathy S., Thapa P., Sivakami M. (2024). Perinatal Depression and Its Associated Risk Factors During the COVID-19 Pandemic in Low- and Middle-Income Countries: A Systematic Review and Meta-Analysis. Soc. Psychiatry Psychiatr. Epidemiol..

[23] White L.K., Kornfield S.L., Himes M.M., Forkpa M., Waller R., Njoroge W.F.M., Barzilay R., Chaiyachati B.H., Burris H.H., Duncan A.F. (2023). The Impact of Postpartum Social Support on Postpartum Mental Health Outcomes During the COVID-19 Pandemic. Arch. Womens Ment. Health.

[24] Mirbahaeddin E., Chreim S. (2024). Transcending Technology Boundaries and Maintaining Sense of Community in Virtual Mental Health Peer Support: A Qualitative Study With Service Providers and Users. BMC Health Serv. Res..

[25] Alshowkan A., Shdaifat E., Alnass F.A., Alqahtani F.M., AlOtaibi N.G., AlSaleh N.S. (2023). Coping Strategies in Postpartum Women: Exploring the Influence of Demographic and Maternity Factors. BMC Womens Health.

[26] Khademi K., Kaveh M.H. (2024). Social Support as a Coping Resource for Psychosocial Conditions in Postpartum Period: A Systematic Review and Logic Framework. BMC Psychol..

[27] Vigod S.N., Tarasoff L.A., Bryja B., Dennis C.-L., Yudin M.H., Ross L.E. (2013). Relation Between Place of Residence and Postpartum Depression. CMAJ.

[28] Abrams L.S., Curran L. (2009). “And You’re Telling Me Not To Stress?” A Grounded Theory Study of Postpartum Depression Symptoms Among Low-Income Mothers. Psychol. Women Q..

[29] Miyake Y., Tanaka K., Sasaki S., Hirota Y. (2011). Employment, Income, and Education and Risk of Postpartum Depression: The Osaka Maternal and Child Health Study. J. Affect. Disord..

[30] Nelson H.D., Darney B.G., Ahrens K., Burgess A., Jungbauer R.M., Cantor A., Atchison C., Eden K.B., Goueth R., Fu R. (2022). Associations of Unintended Pregnancy With Maternal and Infant Health Outcomes: A Systematic Review and Meta-Analysis. JAMA.

[31] Suarez A., Shraibman L., Yakupova V. (2023). Long-Term Effects of Maternal Depression During Postpartum and Early Parenthood Period on Child Socioemotional Development. Children.

[32] Choi Y., Bishai D., Minkovitz C.S. (2009). Multiple Births Are a Risk Factor for Postpartum Maternal Depressive Symptoms. Pediatrics.

[33] Highet N., McCarthy M.J., Lally M.F. (2022). Multiple Birth Mental Health Outcomes Throughout Pregnancy, Delivery and Postnatally. Women Birth.

[34] Thompson L.N., Leistikow N., Smith M.H., Standeven L.R. (2024). The Relationship Between Infant Feeding and Maternal Mental Health: Clinical Vignette. Adv. Psychiatry Behav. Health.

[35] World Health Organization (WHO) (2022). Guide for Integration of Perinatal Mental Health in Maternal and Child Health Services.

[36] Zhang Y., Razza R. (2022). Father Involvement, Couple Relationship Quality, and Maternal Postpartum Depression: The Role of Ethnicity Among Low-Income Families. Matern. Child Health J..

[37] Pebryatie E., Paek S.C., Sherer P., Meemon N. (2022). Associations Between Spousal Relationship, Husband Involvement, and Postpartum Depression Among Postpartum Mothers in West Java, Indonesia. J. Prim. Care Community Health.

[38] Agrawal I., Mehendale A.M., Malhotra R. (2022). Risk Factors of Postpartum Depression. Cureus.

[39] Zhang X., Ma P., Li M. (2023). The Association Between Paternal Childcare Involvement and Postpartum Depression and Anxiety Among Chinese Women-A Path Model Analysis. Arch. Womens Ment. Health.

